# Efficacy of tumour-treating fields therapy in recurrent glioblastoma: A narrative review of current evidence

**DOI:** 10.1097/MD.0000000000036421

**Published:** 2023-12-01

**Authors:** Gbolahan Olatunji, Nicholas Aderinto, Temiloluwa Adefusi, Emmanuel Kokori, Olumide Akinmoju, Ismaila Yusuf, Tobi Olusakin, Muhammad Ali Muzammil

**Affiliations:** a Department of Medicine and Surgery, University of Ilorin, Ilorin, Nigeria; b Department of Medicine and Surgery, Ladoke Akintola University of Technology, Ogbomoso, Nigeria; c Bowen University Teaching Hospital, Ogbomoso, Nigeria; d College of Medicine, University of Ibadan, Ibadan, Nigeria; e Department of Medicine and Surgery, Obafemi Awolowo University, Ife, Nigeria; f Dow University of Health Sciences, Karachi, Pakistan.

**Keywords:** cancer, neurooncology, recurrent glioblastoma, TTFields

## Abstract

Recurrent Glioblastoma presents a formidable challenge in oncology due to its aggressive nature and limited treatment options. Tumour-Treating Fields (TTFields) Therapy, a novel therapeutic modality, has emerged as a promising approach to address this clinical conundrum. This review synthesizes the current evidence surrounding the efficacy of TTFields Therapy in the context of recurrent Glioblastoma. Diverse academic databases were explored to identify relevant studies published within the last decade. Strategic keyword selection facilitated the inclusion of studies focusing on TTFields Therapy’s efficacy, treatment outcomes, and patient-specific factors. The review reveals a growing body of evidence suggesting the potential clinical benefits of TTFields Therapy for patients with recurrent Glioblastoma. Studies consistently demonstrate its positive impact on overall survival (OS) and progression-free survival (PFS). The therapy’s safety profile remains favorable, with mild to moderate skin reactions being the most commonly reported adverse events. Our analysis highlights the importance of patient selection criteria, with emerging biomarkers such as PTEN mutation status influencing therapy response. Additionally, investigations into combining TTFields Therapy with other treatments, including surgical interventions and novel approaches, offer promising avenues for enhancing therapeutic outcomes. The synthesis of diverse studies underscores the potential of TTFields Therapy as a valuable addition to the armamentarium against recurrent Glioblastoma. The narrative review comprehensively explains the therapy’s mechanisms, clinical benefits, adverse events, and future directions. The insights gathered herein serve as a foundation for clinicians and researchers striving to optimize treatment strategies for patients facing the challenging landscape of recurrent Glioblastoma.

## 1. Introduction

Glioblastoma (GBM), formerly known as glioblastoma multiforme, is one of the most aggressive forms of adult gliomas.^[1]^ It originates from primary neoplasms within glial cells or their precursor cells in the central nervous system, constituting approximately 16% of all primary brain and central nervous system tumors.^[2]^ With an age-adjusted incidence rate of 3.2 per 100,000 population,^[3,4]^ glioblastomas predominantly emerge within the frontal, temporal, parietal, and occipital lobes, encompassing a majority share of 61% among all primary gliomas.^[5]^ Although typically manifesting around the median age of 64 years,^[6]^ glioblastomas, including the pediatric population, affect a broad spectrum of ages. The male-to-female ratio stands at 1.6:1, demonstrating a slight predilection among Caucasians compared to other ethnic groups.^[7]^ Glioblastomas are classified as primary or secondary tumors, with primary GBMs arising de novo without precursor lesions.^[8]^ In contrast, secondary GBMs evolve from lower-grade tumors.^[8]^ The former subtype primarily targets the elderly demographic and is associated with a less auspicious prognosis than their secondary counterparts.^[8]^

Genomic exploration has entered a transformative era, exemplified by initiatives like the Cancer Genome Atlas project, which reveals a complex genomic landscape.^[9]^ Sequencing over 600 genes in 200 tumor samples has identified three pivotal signaling pathways – the p53 pathway, the receptor tyrosine kinase/Ras/phosphoinositide 3-kinase pathway, and the retinoblastoma pathway – as recurrently activated culprits.^[10]^ These molecular aberrations drive unchecked cellular proliferation, enhanced cell survival, and evasion from critical cellular checkpoints.^[9,10]^ In response to this genomic data, the World Health Organization (WHO) Classification of Central Nervous System Tumors has redefined GBM stratification based on isocitrate dehydrogenase (IDH) mutation status.^[11]^ This reclassification aligns with distinct genetic trajectories, patient demographics, and treatment responses attributed to IDH-wildtype and IDH-mutant glioblastomas.^[12]^

Within the context of treatment responses, the prominence of recurrent Glioblastoma is distinctly evident.^[12]^ The inherent aggressive behavior and resistance to conventional therapeutic approaches underscore a clinically demanding scenario.^[12]^ In light of this, the imperative to delve into innovative therapeutic strategies has gained substantial momentum, driven by enhancing patient outcomes and elevating their quality of life. Amidst this, Tumor Treating Fields (TTFields) Therapy emerges as a compelling narrative. TTFields, also known as alternating electric fields, oscillate within 100 to 500 kHz frequency and intensities of 1 to 3 V/cm, imposing a nuanced antimitotic rhythm on cellular processes.^[13]^ Preclinical studies reveal the intricate impact of TTFields on cellular processes, positioning it as an innovative therapy.^[13–15]^ Rigorous clinical trials resulted in Food and Drug Administration endorsement of TTFields Therapy for both recurrent and newly diagnosed GBMs.^[16–20]^ Recognition from the American Society of Clinical Oncology and integration into National Comprehensive Cancer Network guidelines highlight its emergence as a promising therapeutic avenue.^[21,22]^ In this review, we critically analyze the evidence to contribute to understanding TTFields Therapy as a promising avenue in the battle against recurrent Glioblastoma. By offering insights into its potential impact within this complex clinical scenario, we endeavor to inform clinical decision-making and inspire further research efforts to advance the treatment outcomes for patients grappling with recurrent Glioblastoma.

## 2. Methodology

Our review explored diverse academic databases, including PubMed, MEDLINE, Scopus and Google Scholar – Table 1. Employing a strategic selection of keywords such as “recurrent glioblastoma,” “Tumor-Treating Fields Therapy,” “TTFields efficacy,” and “glioblastoma treatment,” we identified studies involving TTFields Therapy and the recurrence of Glioblastoma.

**Table 1:** Methodology of review on tumour treating fields (TTFields) therapy for recurrent glioblastoma.

Methodology	Details
Databases searched	PubMed, MEDLINE, Scopus, Google Scholar
Keywords used	“recurrent glioblastoma,” “Tumour-Treating Fields Therapy,” “TTFields efficacy,” “glioblastoma treatment”
Inclusion criteria	- Focus on the efficacy of TTFields Therapy in recurrent glioblastoma - Provision of relevant treatment outcomes - Involvement of human subjects - Publication in English language
Exclusion criteria	- Studies not specifically addressing recurrent glioblastoma - Preclinical studies
Data extraction	Utilized a structured template for extracting critical information
Extracted information	- Study design - Characteristics of patients under study - Treatment protocol details - Reported outcomes (e.g., progression-free survival, overall survival, impact on quality of life) - Adverse effects attributed to TTFields Therapy
Reporting approach	Findings and insights reported in accordance with established reporting guidelines for narrative reviews

TTFields = Tumor-Treating fields.

Our search was confined to studies published within the last decade to ensure the inclusion of contemporary insights. Studies that adhered to the following criteria were considered for inclusion: a focus on the efficacy of TTFields Therapy in the context of recurrent Glioblastoma, provision of relevant treatment outcomes, involvement of human subjects, publication in the English language, and peer-reviewed status. In line with the defined scope, studies that did not align with these criteria, including those of preclinical trials or those not specifically addressing recurrent Glioblastoma, were deliberately excluded.

A structured template facilitated the extraction of pertinent data from the selected studies. Critical information encompassed the study design, characteristics of the patients under study, specifics of the treatment protocol, reported outcomes encompassing parameters like progression-free survival, overall survival, and the impact on quality of life, and documentation of any reported adverse effects attributed to TTFields Therapy. The findings and insights from this review are faithfully reported in congruence with established reporting guidelines for narrative reviews.

## 3. Overview of recurrent glioblastoma

Recurrent Glioblastoma (rGBM), a formidable subset of central nervous system cancer, predominantly affects adults and is most commonly observed in individuals with an average age of 65 years.^[23]^ Despite considerable strides in medical understanding and therapeutic interventions, the prognosis for newly diagnosed GBM patients remains disheartening, with median overall survival ranging from 12 to 18 months and a notably low 5-year survival rate below 7%.^[24,25]^ The formidable nature of rGBM becomes starkly evident as available treatment options remain limited and its prognosis remains suboptimal.^[25]^ With the emergence of tumors after first-line therapy, rGBM presents a complex challenge, characterized by a median progression-free survival spanning 1.5 to 6 months and a median overall survival ranging from 2 to 9 months following recurrence.^[26]^

The therapeutic landscape and prognosis of rGBM are deeply intertwined with its molecular characteristics. Among these, MGMT promoter methylation is significant for prognostication and prediction.^[27]^ Irrespective of the treatment modality, GBM patients harboring a methylated MGMT promoter exhibit improved responses to alkylating chemotherapy and enjoy prolonged survival.^[28]^ Importantly, the influence of MGMT promoter methylation extends to the prognosis during tumor recurrence, with patients bearing methylated rGBMs demonstrating slightly enhanced post-progression survival.^[28]^ Despite the shared features between primary and recurrent glioblastomas, most mutations in well-established cancer-associated genes persist upon recurrence, resulting in remarkable similarities in the genomic landscape between initial GBMs and their recurrent counterparts.^[28]^ However, individual genes exhibit considerable variations, exemplified by the heightened retention of mutations in the TERT promoter and the comparatively diminished retention in the EGFR gene.^[29]^ While hypermutated GBMs, characterized by inactivating mutations in DNA damage repair genes, are relatively rare in rGBM cases,^[30]^ their clinical relevance remains enigmatic, and the impact of alkylating chemotherapy on such tumors appears limited.^[30]^

The formidable challenge of rGBM is further compounded by the emergence of drug-related side effects, as exemplified by the phenomenon of lomustine-induced thrombocytopenia. This adverse effect necessitates therapeutic adjustments, including dose reductions, delays, or even discontinuation, potentially influencing the course of treatment.^[31]^ Thrombocytopenia poses a significant drawback in the therapeutic regimen, impeding effective lomustine administration and compromising treatment efficacy. Research by Jakobsen underscores that thrombocytopenia is particularly notable among patients receiving lomustine combined with bevacizumab, highlighting the necessity of judicious medication adjustments, especially in malignancies featuring MGMT promoter methylation. Individuals undergoing altered treatment due to thrombocytopenia experienced inferior progression-free survival (PFS).^[31]^ The connection between thrombocytopenia and PFS prompts critical inquiries into the treatment’s effectiveness within this specific patient subset, even if overall survival (OS) remains relatively unaffected. Furthermore, the combination of lomustine and bevacizumab amplifies the risk of thrombocytopenia, potentially leading to more frequent discontinuation of lomustine treatment.

Resort to re-resection presents a potential avenue for alleviating symptoms and providing tissue for molecular analysis, although its impact on overall survival remains uncertain.^[32]^ While exploring higher radiation doses is feasible, concerns surrounding adverse effects such as radiation necrosis arise due to the limited tolerance of healthy brain tissue to radiation.^[33]^ Chemotherapeutic possibilities are constrained during recurrence, with the likelihood of an objective response being minimal. Despite bevacizumab, an anti-angiogenic agent, conferring certain benefits in progression-free survival, it does not significantly extend overall survival.^[34]^ Ongoing clinical trials are investigating approaches, including immunotherapy, targeted therapy, and manipulation of the blood-brain barrier, but meticulous investigations are imperative to ascertain their effectiveness.^[35,36]^

The introduction of Optune, a device delivering tumor-treating fields (TTFields), emerges as a promising intervention for recurrent GBM when employed either in isolation or in conjunction with TMZ [^[37]^ 133]. TTFields, characterized by low-intensity alternating electric fields, disrupt cell division, culminating in cell death. In the context of recurrent GBM, Optune has improved survival and fewer adverse effects than conventional treatment.^[37]^ Nevertheless, long-term survival rates are disheartening despite these advancements, and effective treatments for recurrent GBM remain elusive.

REG, a tyrosine kinase inhibitor, showcases potential anti-cancer properties and might outperform existing therapeutic options like LOM, although the data supporting this assertion remain uncertain.^[38]^ REG’s true efficacy and safety profile in glioblastoma treatment remain enigmatic due to the dearth of high-certainty evidence and the constrained patient cohort in relevant studies, some hinting at contradictory outcomes.^[39]^

The investigational drug ABT414 holds promise for potentially surpassing conventional therapy, yet the current evidence’s certainty remains restricted, warranting further meticulous investigations to establish efficacy and safety.^[40]^ On the other hand, Cediranib, another targeted therapy inhibiting angiogenesis, appears to exhibit diminished efficacy compared to conventional therapy and offers marginal additional advantages in combination.^[41]^ Such limitations cast Skepticism on its role as a definitive glioblastoma treatment reminiscent of BEV. Numerous pioneering therapies, including imatinib, axitinib, personalized peptide immunization, nivolumab with or without ipilimumab, pembrolizumab, enzastaurin, and afatinib, have been the subject of scrutiny for glioblastoma treatment.^[42–44]^ However, the evidence substantiating their efficacy remains insufficient, particularly in recurrent Glioblastoma, failing to demonstrate substantial therapeutic benefits. Therefore, the necessity of further research into these therapies may warrant critical examination.

The prevailing paucity of robust evidence and the lack of clarity surrounding multiple treatment modalities underscores the need for rigorous, controlled trials to identify more effective, targeted therapeutic options for glioblastoma patients. Given the heterogeneity of Glioblastoma and its intricate resistance mechanisms, formulating effective treatments poses formidable challenges, emphasizing the imperative of a tailored approach to treatment for augmenting patient outcomes. Addressing these constraints and unraveling efficient, targeted therapeutic strategies is pivotal to enhancing the prognosis for glioblastoma patients and refining the overall treatment landscape.

Tumour Treating Fields (TTFields)‘s profound significance in addressing recurrent Glioblastoma. These fields induce the formation of clusters of cytosolic micronuclei, attracting DNA sensors like cGAS (cyclic GMP-AMP synthase) and AIM2 (absent in melanoma 2).^[43]^ These clusters, arising from disturbances during cell division induced by TTFields, encompass substantial naked micronuclei within the cytosol, extending from the authentic nuclei through focal, narrow bridges. This striking phenomenon consistently emerges in various GBM cell lines upon exposure to TTFields.^[44]^

The DNA sensors, such as cGAS and AIM2, can detect the clusters of cytosolic micronuclei generated by TTFields. These DNA sensors activate their corresponding inflammasomes, cGAS/STING and AIM2/caspase 1, upon recruitment to these clusters.^[45]^ This activation triggers an immunological response and the release of danger signals. At sites where cytosolic micronuclei cluster protrusions occur, TTFields impact the integrity of the nuclear envelope.^[18]^ These clusters might not signify chromosomal condensation during prometaphase, as the theory suggests that focal rupture and perforations result from nuclear envelope disruption. The TTFields-induced nuclear envelope breakdown and the formation of cytosolic micronuclei clusters necessitate entry into the S-phase. In GBM and other cancer cells, TTFields also stimulate the cGAS/STING and AIM2/caspase 1 inflammasome. This activation leads to the upregulation of pro-inflammatory cytokines (PICs), type 1 interferons (T1IFNs), and T1IFN-responsive genes (T1IRGs). This activation is contingent on both STING and AIM2.

## 4. Mechanisms of action of tumor-treating fields therapy

**Figure 1.**
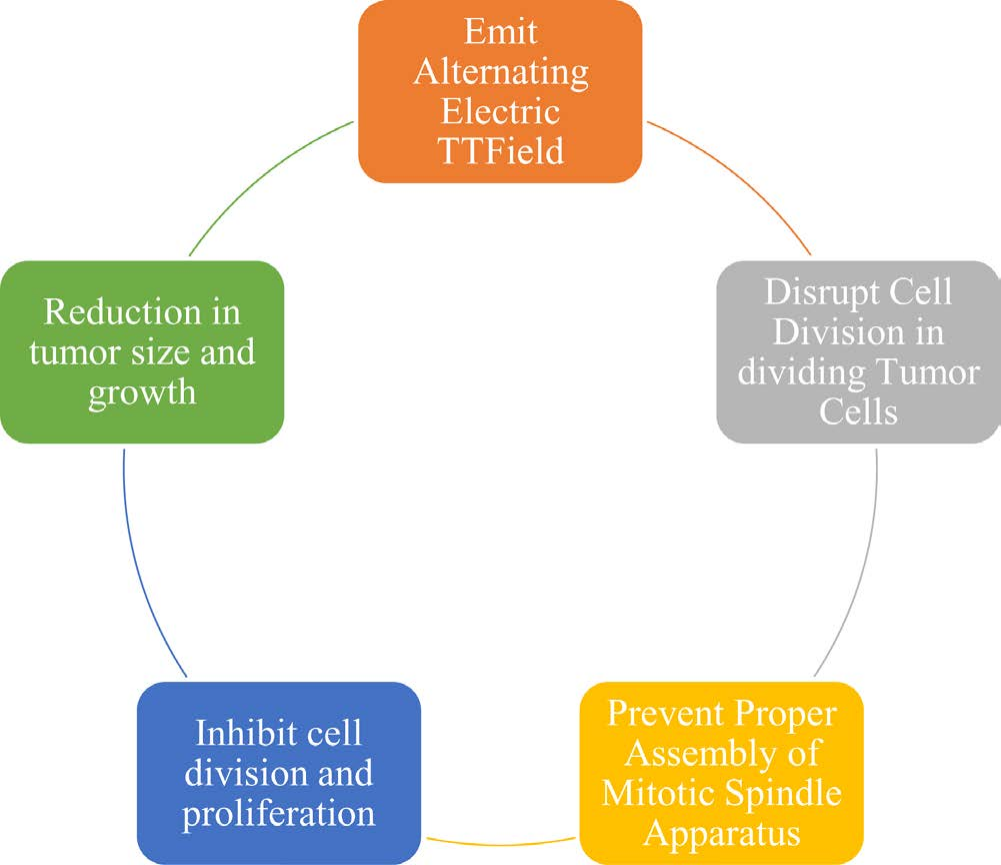
Mechanism of action of tumor-treating fields therapy in recurrent glioblastoma.

Tumour-treating fields (TTFields) therapy, also known as alternating electric field therapy, has firmly established itself as a treatment option for specific types of solid tumors, prominently Glioblastoma^[18]^ – Figure 1. As a safe and noninvasive approach, it has demonstrated remarkable efficacy against solid tumors.^[18]^ Its acceptance and effectiveness were underscored by its Food and Drug Administration approval in 2015 for glioblastoma treatment following the EF-14 trial.^[46]^ This pivotal trial compared TTFields therapy + standard treatment against standard treatment alone, revealing that incorporating TTFields therapy extended median overall survival by 4.9 months and concurrently enhanced overall quality of life. Notably, there was no substantial rise in the rate of systemic adverse effects, with rates being 48% for TTFields therapy and 44% without it.^[47]^

TTFields therapy represents a pioneering strategy for addressing solid tumors, including Glioblastoma, by targeting rapidly dividing cells.^[48]^ It delivers low-intensity, alternating electric currents to the brain via specialized medical devices.^[48]^ While alternating electric currents were widely thought to lack biological impact decades ago, the last decade has unveiled their ability to exert biological effects within cells. The crux of this approach hinges on the heightened susceptibility of tumor cells, characterized by rapid division, to disruptions in the cell division process.^[48]^ The applied electrical current precisely exerts a mechanical force on cellular components, instigating alterations in mitosis and eventual tumor cell demise.^[48]^

TTFields therapy consists of two principal components: the delivery system and the frequency and intensity. The delivery system, exemplified by NovoTTF 200A, encompasses two core elements: insulated transducer arrays and the electric field generator. These insulated transducer arrays, housing two perpendicular electrodes, enable the transcutaneous delivery of electric current.^[49]^

The efficacy of TTFields therapy is contingent on the specific intensity and frequency of the electric fields applied. For treating Glioblastoma, a frequency of 20KHz is employed, whereas a frequency of 150KHz is utilized for managing malignant melanoma. Importantly, these targeted frequencies prevent heat generation, nerve stimulation, and muscle stimulation, thus minimizing the risk of adverse effects post-therapy.^[18]^ The application of alternating electric fields occurs transcutaneously by placing two electrodes on the skin’s surface. The electric field’s magnitude is directly proportional to the potential difference between the two electrodes and inversely proportional to the distance between them.^[50]^

The mechanism of action of TTFields therapy is rooted in two fundamental principles: dipole alignment and dielectrophoresis.^[44,51]^ The principle of dipole alignment capitalizes on the existence of negatively and positively charged proteins within cells that orient towards the oppositely charged electrodes when exposed to an electric field. Additionally, the concept of dielectrophoresis operates on the movement of molecules towards regions of heightened electric field intensity when subjected to electric current.^[51]^

These combined principles underpin the foundation of TTFields therapy.^[51]^ Studies have consistently demonstrated that TTFields therapy functions by disrupting microtubules and impeding mitosis, inducing cellular fragmentation, promoting apoptosis in cancer cells,^[52]^ and augmenting the permeability of the blood-brain barrier.^[51–53]^ Notably, the enhanced permeability of the blood-brain barrier has been associated with the observed efficacy of TTFields therapy when integrated into standard treatment, surpassing the outcomes of standard treatment alone [^[54]^ 148].

TTFields therapy exclusively targets cells in the division phase.^[52]^ The alternating electric field generates dielectrophoretic forces that impair the normal functioning of microtubules, cilia, and mitotic spindles. This collective disruption results in the impairment of the overall mitotic process, leading to the failure of cancer cell proliferation.^[52]^ According to Giladi et al,^[52]^ TTFields therapy heightens tubulin depolymerization, disrupts conventional microtubule assembly, and attenuates DNA replication.

The inhibitory impact of TTFields therapy culminates in the death of tumor cells, triggering a reduction in tumor mass over time and ultimately resulting in improved patient outcomes.^[46]^ TTFields therapy achieves optimal efficacy against rapidly dividing tumor cells.^[47]^ Nevertheless, cell division alteration is not the sole mechanism influencing tumor cell growth. Chang et al^[47]^ highlighted that TTFields therapy increases the permeability of glioblastoma cells. Their study revealed an escalation in the number of membrane pores in glioblastoma cells using scanning electron microscopy.

Furthermore, they demonstrated increased ingress of membrane-associated molecules. Significantly, these changes were specific to cancer cells, as evidenced by their absence in fibroblast cells subjected to similar conditions. Furthermore, the reversibility of these changes was evident, with cessation observed 24 hours after discontinuation of TTFields therapy.^[50]^

While the primary mechanism of TTFields therapy involves cell division alterations, evidence suggests its effects extend to the tumor microenvironment. Notably, the components of the tumor microenvironment affected by TTFields therapy include angiogenesis, immune response, and increasing membrane permeability.^[54,55]^ TTFields therapy thwarts angiogenesis, a malignancy hallmark, which reduces the tumor’s blood and nutrient supply, ultimately causing shrinkage and apoptosis.^[54]^ Moreover, TTFields therapy amplifies the anti-cancer immune response by elevating the display of calreticulin on cell surfaces.^[55]^ Additionally, TTFields therapy has been shown to augment membrane permeability in glioblastoma cells, facilitating the ingress of membrane-penetrating agents like ethidium D and 5-aminolevulinic acid (5-ALA). It also bolsters the blood-brain barrier, enhancing chemotherapy brain absorption.^[53]^ Furthermore, TTFields therapy diminishes the proportion of DNA damage repair by reducing ataxia telangiectasia mutated (ATM) levels and curtailing the capacity of cancer cells to repair DNA damage.^[55]^

## 5. Current evidence on the efficacy of TTFields therapy in recurrent glioblastoma

The studies presented in this review collectively provide a comprehensive assessment of the efficacy of TTFields as a novel treatment modality for rGBM – Table 2. TTFields exert their effects by emitting alternating electric fields, offering a unique approach to tackling the challenges posed by this aggressive cancer. Across the various studies, consistent evidence emerges regarding the potential clinical benefits of TTFields therapy in terms of OS and PFS for patients with rGBM.

**Table 2:** Summary of Key Studies Evaluating Efficacy of TTFields Therapy in Recurrent Glioblastoma.

Authors and Year	Methods	Participants	Positive outcomes	Negative/adverse outcomes	Conclusion
Kesari et al, [2017]	Investigated TTFields + chemotherapy vs chemotherapy alone in recurrent GBM after first recurrence.	203	Longer median OS in TTFields + chemo group	Low toxicity profile; no grade 3/4 device-related adverse events	TTFields + chemotherapy prolongs OS in recurrent GBM after first recurrence.
Mikic, N., et al [2021]	Evaluated skull remodeling surgery + TTFields + best oncology treatment vs TTFields + best oncology treatment in first recurrence GBM.	70	Safety and feasibility demonstrated; potential to improve outcome.	None	Skull remodeling + TTFields + oncology treatment is safe and holds potential to improve outcome in GBM.
Kanner et al, [2014]	Compared NovoTTF therapy vs best physician’s choice chemotherapy in recurrent GBM.	237	Higher median OS with NovoTTF therapy	Mild to moderate skin rash beneath transducer arrays (14%)	NovoTTF therapy provides OS benefit in recurrent GBM.
Korshoej, A. R., et al [2020]	Tested safety and feasibility of skull remodeling surgery + TTFields + oncology treatment in recurrent GBM.	15	Increased TTFields dose with skull remodeling surgery	71 adverse events (grades 1-3), no grade 4/5 or intervention-related serious AEs	Skull remodeling + TTFields + oncology treatment is safe and holds potential to improve outcome in GBM.
Mrugala, M. M., et al [2014]	Investigated NovoTTF therapy in recurrent GBM patients in real-world clinical practice.	457	Longer median OS in NovoTTF therapy group	Mild to moderate skin rash beneath transducer arrays (14%)	NovoTTF therapy offers clinical benefit and favorable safety profile in real-world setting.
Stupp, R., et al [2012]	Evaluated NovoTTF-100A vs active chemotherapy in recurrent GBM.	237	Comparable efficacy to chemotherapy; mild to moderate skin rash (14-16%)	NovoTTF therapy provides efficacy comparable to chemotherapy, with quality of life benefits.	TFF therapy is effective
Wong et al, [2015]	Explored NovoTTF-100A + bevacizumab vs TCCC + bevacizumab in recurrent GBM.	37	Trend for prolonged OS with NovoTTF + TCCC combination	Mild to moderate skin rash beneath transducer arrays	Combining NovoTTF-100A and TCCC may improve survival.
Dono et al, [2021]	Investigated TBI regimen vs BBC + T treatment in recurrent glioblastoma patients.	48	Improved median OS (10.3 vs 4.1 months) with TBI regimen	Grade III hypertension (38.9%), leukopenia (22.2%)	TBI regimen may improve survival and response in recurrent glioblastoma.
Dono, A., et al [2021]	Investigated impact of PTEN mutation on TTFields therapy in recurrent glioblastoma.	29	Significant PPS benefit in PTEN-mutant rGBM with TTFields	None	PTEN mutation status may influence response to TTFields therapy.

GBM = Glioblastoma, OS = overall survival, PPS = progression-free survival, PTEN = Phosphatase and Tensin Homolog, TBI = Temozolomide, Bevacizumab, and Irinotecan, TCCC = Triple Chemotherapy Combination, TTFields = Tumor-Treating Fields.

### 5.1. Tumour treating fields (TTFields) efficacy

The work of Kesari et al^[56]^ contributes crucial insights through a post hoc analysis of the EF-14 trial, revealing that the combination of TTFields and chemotherapy after initial recurrence results in prolonged OS among rGBM patients. Significantly, this therapeutic approach is accompanied by a low toxicity profile, with no severe device-related adverse events reported. Similarly, Mikic et al^[57]^ explore the integration of skull remodeling surgery, TTFields, and medical oncological therapy in the OptimalTTF-2 trial. This study successfully validates the feasibility and safety of this multifaceted intervention in a phase I context, underscoring its potential to yield improved treatment outcomes.

The significance of patient compliance with TTFields therapy is highlighted in the analysis by Kanner et al,^[58]^ where patients with high adherence to TTFields experience markedly improved median OS compared to those undergoing traditional chemotherapy.

The study by Korshoej et al^[59]^ introduces the idea of merging TTFields with skull remodeling surgery, emphasizing its safety and lack of toxicity. This innovative approach demonstrates feasibility and raises the potential for localized dose enhancement, possibly improving treatment outcomes. Mrugala et al^[60]^ contribute a real-world perspective by analyzing the Patient Registry Dataset, reinforcing the clinical benefits of NovoTTF Therapy in rGBM. The analysis showcases improved survival rates compared to earlier trials, thus solidifying the therapy’s efficacy in real-world clinical settings.

The pivotal phase III trial conducted by Stupp et al^[40]^ underscores the comparable efficacy of NovoTTF Therapy to conventional chemotherapy regimens for rGBM. Importantly, this trial underscores the therapy’s advantageous toxicity profile and its potential to enhance the quality of life for patients. The exploration of combined therapies takes center stage in the study by Wong et al,^[61]^ suggesting that augmenting NovoTTF-100A with a multi-drug regimen could potentially yield enhanced responses among patients with recurrent GBM.

### 5.2. Combination therapies and novel approaches

Combining Tumor Treating Fields (TTFields) with other therapies and innovative interventions emerges as a recurring motif across the reviewed studies, reflecting a collective effort to optimize treatment strategies for recurrent Glioblastoma (rGBM). Mikic et al^[57]^ present a novel approach in the OptimalTTF-2 trial, proposing integrating skull remodeling surgery, TTFields, and medical oncological therapy. This innovative strategy is rooted in preclinical evidence suggesting a synergistic interplay between surgical intervention and the application of TTFields, aiming to enhance treatment outcomes. Similarly, Wong et al^[61]^ explore the combination of NovoTTF-100A with a multi-drug regimen to orchestrate an anti-tumor immunologic response. This innovative approach seeks to extend survival and enhance treatment responses among patients with recurrent GBM.

Throughout these explorations of combination therapies and novel interventions, a common thread is the consideration of potential adverse events associated with these multi-modal approaches. Mikic et al^[57]^ present the OptimalTTF-2 trial, where integrating skull remodeling surgery with TTFields and medical oncological treatment is safe and nontoxic. This encouraging finding provides valuable insights into the feasibility of multi-modal approaches that synergise the benefits of different therapeutic modalities.

### 5.3. Patient selection and biomarkers

The studies collectively delve into patient-specific considerations and the safety profile of TTFields therapy, shedding light on factors impacting treatment response and patient well-being. Patient selection and biomarkers emerge as key topics, with Dono et al^[62]^ investigating the influence of Phosphatase and Tensin Homolog Deleted (PTEN) mutation on the response to TTFields therapy in recurrent Glioblastoma Rgbm. The study presents the intriguing possibility of using PTEN mutation status as a predictive biomarker for therapy outcomes, potentially enhancing personalized treatment approaches.

### 5.4. Adverse events and toxicity

While TTFields therapy is generally associated with low toxicity, the studies acknowledge the presence of adverse events that, albeit generally mild to moderate, warrant consideration. Kesari et al^[56]^ emphasize the low toxicity profile of TTFields therapy, with no occurrence of grade 3/4 device-related adverse events. Similarly, Mrugala et al^[60]^ underscore the absence of unexpected adverse events, highlighting the manageable nature of the therapy’s side effects.

Mild to moderate skin reactions or rashes consistently emerge as recurring adverse events linked to TTFields therapy across the reviewed studies. Although generally manageable and not severe, these skin reactions can contribute to discomfort for patients undergoing treatment. Stupp et al (2012) and Mrugala et al (2014) both acknowledge the presence of mild to moderate skin reactions associated with applying NovoTTF Therapy transducer arrays, affirming the importance of addressing patient comfort.^[40,60]^ Throughout the studies, patient tolerance to TTFields therapy remains a positive aspect. Patients commonly report that adverse events, including skin reactions, are manageable and well-tolerated. Mrugala et al^[60]^ reinforce this notion, emphasizing that NovoTTF Therapy is well-tolerated, with skin reactions attributed to the transducer arrays being the most frequent adverse events.

## 6. Comparison with standard therapy or alternative treatment approaches

Comparing Tumor Treating Fields (TTFields) therapy to standard treatment options or alternative approaches reveals promising outcomes in addressing recurrent Glioblastoma (rGBM). TTFields therapy, a novel antimitotic treatment, involves placing transducer arrays on the scalp to emit low-intensity alternating electric fields that disrupt the division of glioblastoma cells and hinder tumor growth.^[63]^

A post-approval registry study (EF-19) investigated TTFields monotherapy (200 kHz) versus physician’s choice standard of care (PC-SOC) for rGBM patients. The study found that in the intent-to-treat group, TTFields monotherapy showed comparable overall survival (OS) outcomes to PC-SOC. However, in the per-protocol group, TTFields monotherapy demonstrated a notably longer median OS than PC-SOC (8.1 vs 6.4 months).^[47]^ These findings suggest that TTFields therapy could be a viable alternative for rGBM patients, offering improved survival rates compared to current treatments.

In the context of newly diagnosed GBM, a randomized clinical trial involving 695 patients explored the combination of TTFields therapy with maintenance temozolomide chemotherapy. Adding TTFields to maintenance temozolomide significantly enhanced progression-free survival (6.7 months vs 4.0 months).^[47]^ These results indicate that combining TTFields therapy with standard chemotherapy might benefit GBM treatment strategies.

Moreover, TTFields therapy has exhibited good tolerance and minimal systemic side effects, making it a suitable option for recurrent glioblastoma patients who might not be eligible for further surgeries or aggressive treatments.^[63]^ While TTFields therapy offers benefits such as non-invasiveness, personalized treatment, and improved survival rates, it also comes with drawbacks related to device usage and patient compliance. The requirement for patients to wear transducer arrays on their heads for extended periods could potentially impact compliance and convenience.^[63]^ Further research is warranted to assess long-term survival outcomes and explore the potential for developing imaging biomarkers to optimize therapy utilization.^[64]^

Tumour Treating Fields (TTFields) therapy has emerged as a promising noninvasive approach in the realm of antimitotic treatments, offering a viable avenue for addressing the formidable challenge posed by recurrent Glioblastoma (rGBM), a devastating and often refractory malignant brain tumor. Compared to alternative treatment modalities, several key advantages and limitations of TTFields therapy warrant comprehensive examination, shedding light on its potential impact on clinical management.

1. **Efficacy:** Extensive clinical investigations have underscored the potential of TTFields therapy to significantly augment both PFS and overall survival (OS) outcomes when combined with adjuvant temozolomide treatment in newly diagnosed glioblastoma (GBM) patients.^[47]^ Moreover, in the realm of recurrent GBM, TTFields therapy has demonstrated comparable effectiveness to standard chemotherapy in terms of OS.^[47]^ Impressively, individualized TTFields monotherapy has even shown the capability to surpass standard care, leading to prolonged patient survival rates.
2. **Non-invasiveness:** One of the paramount advantages of TTFields therapy lies in its noninvasive nature. Unlike conventional surgical procedures or aggressive interventions, TTFields therapy involves the application of controlled electrical fields to the scalp, offering a less burdensome and more patient-friendly treatment approach.^[64]^ This characteristic enhances patient comfort and minimizes the potential for postoperative complications.
3. **Diverse mechanisms of action:** TTFields therapy harnesses a multifaceted mechanism of action, further bolstering its therapeutic potential. Through intricate pathways, TTFields therapy impedes cancer cell proliferation, disrupts DNA repair processes, hinders angiogenesis, stimulates apoptosis and immunogenic cell death, and exhibits pronounced antimitotic effects.^[65]^ This multifunctional approach underscores the robustness of TTFields therapy in targeting various aspects of tumor growth and survival.

### 6.1. Limitations of TTFields therapy

1. **Cost implications:** The economic feasibility of TTFields therapy is a pertinent consideration. The relatively high costs of this innovative treatment approach might pose barriers, particularly in regions with limited healthcare resources.^[66]^ These financial considerations could potentially limit patient access and adherence to the therapy, warranting careful attention.
2. **Adverse effects:** While generally well-tolerated, TTFields therapy is not devoid of adverse effects. Notably, requiring patients to wear transducer arrays on the scalp for extended durations can lead to skin rashes and irritation at the application site.^[67]^ Though manageable, these localized reactions should be considered when evaluating the patient experience.
3. **Variable efficacy:** The efficacy of TTFields therapy might exhibit variability among different patient subgroups. Factors such as age, Karnofsky Performance Status (KPS), O6-methylguanine-DNA methyltransferase (MGMT) methylation status, and the number of tumor recurrences could influence the therapy’s effectiveness.^[67]^ This inherent variability emphasizes the need for personalized treatment strategies based on patient-specific characteristics.

In the dynamic landscape of rGBM treatment, TTFields therapy presents an array of compelling advantages, encompassing its efficacy, non-invasiveness, and multifaceted mechanisms of action. However, several critical aspects warrant careful consideration in contemplating its integration as a therapeutic option. Financial implications, potential adverse effects, and the variability in treatment response underline the importance of a nuanced evaluation when incorporating TTFields therapy into the clinical management of rGBM patients.

Evaluating comparative studies about treating recurrent rGBM is paramount in shaping informed and effective therapeutic decisions. Through a meticulous assessment of diverse treatment methodologies, these studies offer invaluable insights into the most efficacious and suitable approaches for individuals grappling with this challenging condition.

1. **Retrospective multicenter study:** A comprehensive retrospective multicenter study sought to evaluate the efficacy of conventional rGBM treatment modalities, encompassing systemic therapy, re-irradiation, and re-resection followed by adjuvant therapy and optimal supportive care. Remarkably, all patients’ median overall survival was 6.5 months. This study identified age and the presence of multifocal lesions as pivotal factors influencing outcomes, emphasizing the significance of individualized considerations in treatment planning.^[68]^
2. **Bayesian network meta-analysis:** A Bayesian network meta-analysis delved into the effectiveness of various treatment avenues for rGBM, including bevacizumab, temozolomide, lomustine, and regorafenib. Notably, this investigation underscored the importance of active participation in clinical trials before resorting to conventional treatments. The study’s findings underscored the potential benefits of experimental approaches in the context of recurrent GBM.^[69]^
3. **Role of reoperation study:** A retrospective exploration aimed to elucidate the role of reoperation in the context of rGBM. This investigation revealed divergent evidence regarding the prognostic implications of reoperation. Salvage interventions like reoperation exhibited the potential to provide a subset of rGBM patients with meaningful therapeutic benefits, introducing a nuanced dimension to treatment decisions.^[70]^

While these studies provide valuable insights, it is essential to recognize the inherent limitations of retrospective analyses, including potential selection biases and methodological constraints. To robustly guide treatment decisions, rigorous data stemming from well-designed clinical trials are indispensable. Rigorous investigations focusing on comparative efficacy, safety profiles, and improvements in quality of life are imperative in navigating the complex treatment landscape of rGBM. Collaboration among neuro-oncology institutions emerges as a pivotal avenue for elucidating optimal therapeutic strategies, quintessential for addressing the pressing needs of patients grappling with the formidable challenges posed by this aggressive and lethal disease.^[71]^

## 7. Safety and tolerability of TTFields therapy

Assessing safety and adverse events linked to TTFields therapy is paramount to understanding its viability as a treatment modality. TTFields therapy, a noninvasive approach employing electric fields to disrupt crucial cellular processes in cancer cells, has gained approval for newly diagnosed Glioblastoma, recurrent Glioblastoma, and pleural mesothelioma, with investigations extending to other cancer types.

A prospective trial focusing on Japanese patients with newly diagnosed Glioblastoma demonstrated favorable tolerability of TTFields therapy, with no discernible treatment-limiting toxicities.^[72]^ Similarly, a study encompassing adult patients with newly diagnosed Glioblastoma unveiled comparable rates of grade 3 adverse events to other glioblastoma studies, with manageable skin-related adverse events predominantly observed, posing minimal disruptions to treatment.^[73]^ Exploring TTFields therapy in pediatric patients with malignant brain tumors further affirmed its safety, with limited harmful effects recorded.^[74]^ Insights from experts regarding dermatologic adverse events tied to TTFields therapy highlighted manageable skin events as the most common concern, addressable through topical interventions.^[75]^ TTFields therapy exhibits a favorable safety profile, predominantly characterized by mild-to-moderate skin-related side effects.

Within cancer treatment, considerations encompassing patient tolerance, quality of life implications, and treatment-associated side effects emerge as pivotal determinants. Addressing apprehensions surrounding treatment-induced adverse effects, especially in the context of elderly cancer patients, holds significance during decision-making.^[76]^ The integration of geriatric assessment findings can serve as a prognostic tool for gauging treatment tolerability among individuals aged 65 and above, with patient-reported outcomes and functional status as pivotal markers in enhancing treatment choices.^[76]^ Moreover, recognizing patient preferences and tolerance for chemotherapy side effects can profoundly influence treatment decisions in advanced-stage lung cancer.^[77]^ A patient-centric approach acknowledging individual preferences for side effects can significantly enhance treatment outcomes, advocating the potential utility of tools to facilitate patient chemotherapy preferences in clinical settings. The effective management of treatment-related side effects remains paramount in preserving patients’ quality of life during cancer therapy. In immune checkpoint inhibitors cases, a profound understanding of chronic immune-related adverse events (irAEs) is pivotal for ensuring patient well-being and adherence.^[76]^ Effectively addressing these issues underscores the importance of a comprehensive approach incorporating patient perspectives, ultimately contributing to improved outcomes and experiences.

Incorporating patient viewpoints and preferences into the decision-making process assumes pivotal significance in cancer treatment discussions, particularly in the context of Tumor Treating Fields (TTFields) therapy. As a noninvasive, locoregional therapeutic approach, TTFields therapy has secured approval for application in Glioblastoma (GBM) and malignant pleural mesothelioma.^[78]^ However, due to limited safety data, the current therapeutic label for TTFields does not encompass its use in GBM patients with ventriculoperitoneal (VP) shunts. A post-marketing surveillance study ventured to investigate TTFields therapy’s safety in GBM patients with VP shunts, revealing predominantly minor and localized adverse events primarily linked to skin-related concerns.^[78]^

The potential of TTFields therapy extends to pediatric patients with malignant brain tumors, particularly high-grade gliomas, which present formidable treatment challenges. In light of the limitations of conventional therapies for pediatric Central Nervous System malignancies, TTFields therapy emerges as a noninvasive avenue warranting exploration.^[74]^ TTFields therapy demonstrated efficacy and safety in treating newly diagnosed Glioblastoma in Japanese patients, showcasing its potential as a patient-centered treatment option.^[72]^ Acknowledging and incorporating patient opinions and preferences is pivotal for optimizing the utilization of TTFields therapy. Tailoring treatment approaches in alignment with patient choices can enhance treatment adherence and overall success, emphasizing the importance of personalized care in the oncology landscape.

## 8. Future directions and challenges

The reverberations of the EF-11 and EF-14 trials have significantly shaped the trajectory of TTFields therapy for Glioblastoma, illuminating new avenues and challenges.^[79]^ However, certain intricacies remain enigmatic in this evolving landscape, inviting deeper scrutiny and exploration.

Recent advancements have shed light on the intricate mechanism underlying TTFields therapy. Initially, two main mechanisms were proposed: the disruption of polar tubulin orientation leading to interference in microtubule assembly and cell destruction through the exertion of mechanical forces during mitosis.^[80]^ Nevertheless, subsequent laboratory studies have unveiled additional dimensions, including the disruption of Septin fibers and the effect of electric fields on the endoplasmic reticulum, sparking the process of autophagy.^[81]^ This evolving understanding underscores the multifaceted and nuanced nature of TTFields’ biological action, necessitating further meticulous investigation.^[82,83]^

In the domain of recurrent GBM, the clinical effectiveness of TTFields therapy remains a subject of ongoing debate. The EF-11 trial yielded no significant increase in overall survival between treatment and control groups (6.6 months vs 6 months). However, subsequent studies have reported improved overall survival compared to EF-11 controls. Notably, the Patient Registry Dataset highlighted a median overall survival of 9.6 months. Moreover, dissecting EF-14 trial data uncovered an extended median overall survival of 11.8 months when TTFields were integrated with Temozolomide (TMZ) after the first recurrence, surpassing the 9.2 months observed with TMZ monotherapy.^[84]^ These disparities underscore the need for comprehensive exploration into TTFields’ clinical efficacy in the recurrent GBM context and its potential synergistic partnerships.^[85]^

Unfortunately, patient-specific factors influencing treatment efficacy remain relatively underexplored, necessitating a more thorough investigation.^[56]^

1. **NovoTTF treatment signatures in glioblastoma patients at autopsy**: Initiated in 2017, this observational study enrolling 20 participants delves into the cellular narratives within postmortem brain samples from GBM patients. Seeking to decipher the pathological lexicon of tumor treatment fields, it compares the stories of patients who embarked on TTFields therapy at diagnosis with those who initiated it at recurrence.^[86]^
2. **TTFields and radiosurgery of recurrent glioblastoma +/- 18F-fluoro-ethyl-thyrosine**: Commenced in 2020, this phase II open-label trial involving 40 participants with recurrent GBM explores the interplay between stereotactic radiosurgery and TTFields therapy. This symphonic collaboration aims to elevate treatment outcomes while keeping the undertones of toxicity minimal.^[87]^
3. **Open-label pilot study of OPTUNE® with high-density transducer arrays for the treatment of recurrent GBM**: Launched in 2020, this phase II open-label endeavor, enrolling 25 individuals navigating the terrain of recurrent GBM, aims to uncover safety nuances and traverse the uncharted landscapes of enhanced clinical outcomes. It harnesses the potential of high-intensity transducer arrays, weaving a novel narrative in the continuum.^[88]^
4. **Improving tumor treating fields treatment for brain cancer patients with skull remodeling surgery (Neurosurgery**): Embarked upon in 2020, this phase II interventional odyssey gathers 70 participants at the crossroads of the first recurrence in GBM. The journey tests the hypothesis that a harmonious interplay between TTFields therapy and skull remodeling surgery can amplify the therapy’s efficacy, thus elevating overall survival. The minor ballet of skull remodeling surgery ushers electric fields from the transducer array through paths of least resistance.^[89]^

The global distribution of TTFields therapy is characterized by geographical disparities, with the majority seeking refuge within the embrace of the USA.^[90]^ This mosaic of utilization weaves a tapestry shaped by multifaceted threads.

The financial landscape casts its imposing shadow, influencing the broader canvas of TTFields adoption,^[91]^177]. Novocure, the vanguard of TTFields’ delivery, orchestrates rentals at a monthly fee of $21,000, a figure veiled in opacity over auxiliary expenses.^[92]^ Health insurance systems that embrace TTFields therapy shoulder substantial burdens, engendering hesitancy in its adoption across various nations, resonating even within the chambers of the NHS. The canvas of cost unfolds to €243,141, an opulence eclipsing conventional therapies fourfold.^[92]^

The quest for clarity in TTFields, relative to established standards, triggers hesitance within neuro-oncology. The absence of a placebo arm in the EF-11 narrative was grounded in ethical complexities, as the imposition of spurious devices upon GBM sufferers evoked moral dilemmas.^[92]^ Compliance emerges as a looming specter; the harmony of TTFields therapy crescendos when worn for > 18 hours daily. The challenge of adherence murmurs persistently.^[93]^ Despite amelioration, skin irritations and the breach of personal space stand as sentinels curtailing universal embrace.^[81]^

## 9. Conclusion

The comprehensive narrative review of the efficacy of Tumor-Treating Fields (TTFields) Therapy in the context of rGBM provides a holistic understanding of this innovative therapeutic approach. The amalgamation of diverse studies reveals a growing body of evidence supporting the potential clinical benefits of TTFields Therapy in terms of improved overall survival and progression-free survival for patients facing the formidable challenge of rGBM.

The evidence underscores the importance of patient selection criteria and biomarkers in predicting therapy response. Studies exploring the impact of patient-specific factors, such as PTEN mutation status, provide insights that could shape personalized treatment strategies in the future. Moreover, investigations into combining TTFields Therapy with other treatments, as exemplified by the exploration of surgical interventions and novel combination approaches, offer promising avenues for enhancing therapeutic outcomes and expanding the therapeutic landscape.

The favorable safety profile of TTFields Therapy, characterized by manageable skin reactions, supports its clinical applicability and patient tolerance. The therapy’s compatibility with patients’ quality of life is critical, especially in the rGBM setting, where treatment-related toxicity can impact patients’ well-being.

As the field of oncology continues to evolve, TTFields Therapy stands as a testament to the potential of innovative therapeutic approaches. The synthesis of current evidence underscores the importance of a multidisciplinary approach, combining clinical insights, mechanistic understanding, and patient-centered outcomes. While challenges remain, including refining patient selection criteria and exploring optimal combination strategies, the cumulative evidence strongly suggests that TTFields Therapy holds promise in the battle against rGBM.

In a landscape marked by limited treatment options for rGBM, TTFields Therapy offers a ray of hope. The culmination of research efforts reflected in this review not only sheds light on the therapeutic efficacy of TTFields but also paves the way for continued exploration and advancement in neuro-oncology. As future studies build upon the foundations established here, the potential of TTFields Therapy to reshape the treatment landscape for rGBM becomes increasingly evident, offering renewed optimism for patients, clinicians, and researchers alike.

## Author contributions

**Conceptualization:** Gbolahan Olatunji, Nicholas Aderinto.

**Writing – original draft:** Gbolahan Olatunji, Nicholas Aderinto, Temiloluwa Adefusi, Emmanuel Kokori, Olumide Akinmoju, Ismaila Yusuf, Tobi Olusakin, Muhammad Ali Muzammil.

**Writing – review & editing:** Gbolahan Olatunji, Nicholas Aderinto, Temiloluwa Adefusi, Emmanuel Kokori, Olumide Akinmoju, Ismaila Yusuf, Tobi Olusakin, Muhammad Ali Muzammil.
